# Effect of Bone Marrow Stromal Cells in Parkinson's Disease Rodent Model: A Meta-Analysis

**DOI:** 10.3389/fnagi.2020.539933

**Published:** 2020-12-11

**Authors:** Jianyang Liu, Jialin He, Yan Huang, Zhiping Hu

**Affiliations:** Department of Neurology, Second Xiangya Hospital, Central South University, Changsha, China

**Keywords:** bone marrow stromal cell, Parkinson's disease, meta-analysis, efficacy, animal experimentation

## Abstract

**Background:** Bone marrow stromal cells (BMSCs) has been reported to have beneficial effects in improving behavioral deficits, and rescuing dopaminergic neuron loss in rodent models of Parkinson's disease (PD). However, their pooled effects for dopaminergic neuron have yet to be described.

**Objective:** To review the neuroprotective effect of naïve BMSCs in rodent models of PD.

**Methods:** The PubMed, EMBASE, and Web of Science databases were searched up to September 30, 2020. Inclusion criteria according to PICOS criteria were as follows: (1) population: rodents; (2) intervention: unmodified BMSCs; (3) comparison: not specified; (4) primary outcome: tyrosine hydroxylase level in the substantia nigra pars compacta and rotational behavior; secondary outcome: rotarod test, and limb function; (5) study: experimental studies. Multiple prespecified subgroup and meta-regression analysis were conducted. Following quality assessment, random effects models were used for this meta-analysis.

**Results:** Twenty-seven animal studies were included. The median quality score was 4.7 (interquartile range, 2–8). Overall standardized mean difference between animals treated with naïve BMSCs and controls was 2.79 (95% confidence interval: 1.70, 3.87; *P* < 0.001) for densitometry of tyrosine hydroxylase-positive staining; −1.54 (95% confidence interval: −2.11, −0.98; *P* < 0.001) for rotational behavior. Significant heterogeneity among studies was observed.

**Conclusions:** Results of this meta-analysis suggest that naïve BMSCs therapy increased dopaminergic neurons and ameliorated behavioral deficits in rodent models of PD.

## Introduction

Parkinson's disease (PD) is a neurodegenerative disorder characterized by clinical motor symptoms of bradykinesia, muscle rigidity, tremor, and postural instability (Jankovic, 2008), resulting from the selective degeneration of dopaminergic neurons in the substantia nigra pars compacta (SNpc) (Michely et al., 2015) and intraneuronal protein aggregates called Lewy bodies (Lashuel et al., 2013). As the fastest growing neurodegenerative disease in the world, PD prevalence is projected to exceed 12 million by 2040 (Dorsey et al., 2018). The main therapies of PD include L-3,4-dihydroxyphenylalanine (L-DOPA) dopamine agonists, enzyme inhibitors and deep brain stimulation (Dong et al., 2016). However, the above therapies remain insufficient to recover the massive loss of dopaminergic neurons. Cellular therapy is another novel therapeutic tool that offers considerable hope and promise to promote neural recovery in PD (Lo Furno et al., 2018; Staff et al., 2019). Various source tissues have been tested for efficiency of mesenchymal stem cells therapy for PD, such as human bone marrow (Ye et al., 2007), adipose (Berg et al., 2015; Cucarian et al., 2019), olfactory mucosa (Simorgh et al., 2019), placenta (Kim et al., 2018), umbilical cord (Zhao et al., 2016), umbilical cord blood (Lee et al., 2019), and deciduous teeth (Zhang N. et al., 2018). Among the many kinds of mesenchymal stem cells in preclinical studies, bone marrow-derived mesenchymal stem cells are the most well-tested.

Bone marrow stromal cells (BMSCs, also known as bone marrow-derived mesenchymal stem and progenitor cells) have the potential to differentiate to mesenchymal lineage, such as osteoblasts, chondrocytes, adipocytes, and muscle (Prockop, 1997). BMSCs are easily accessible and isolated through aspiration of the bone marrow. They are free of ethical controversy, and are associated with fewer immunological reactions (Pittenger et al., 1999). They also have the ability to be easily expanded on a large scale, which is very convenient and suitable for clinical use (Dezawa, 2006). BMSCs have the potential to differentiate into functional dopaminergic neurons (Dezawa et al., 2004; Bae et al., 2011; Datta et al., 2011; Venkatesh and Sen, 2017) without forming tumors in preclinical studies (Rengasamy et al., 2016).

Numerous researches have evaluated the efficacy of BMSCs transplantation for PD, yet there are some disputes over results. Prior meta-analysis either took a broader approach to mesenchymal stem cells therapy (Riecke et al., 2015) or only included the induced pluripotent stem cells therapy (Zhang Y. et al., 2018). Neither have offered a meta-analysis of the relevant study to investigate both histopathological and functional efficiency of BMSCs transplantation for PD. Our aim was to perform a meta-analysis to review published animal studies employing the use of naïve BMSCs therapy following PD, and to provide information for the future clinical translation of BMSCs to the bedside.

## Materials and Methods

Preferred Reporting Items for Systematic Reviews and Meta-Analysis (PRISMA) was used to perform this meta-analysis (Moher et al., 2009).

### Search Strategy

Studies of bone marrow-derived mesenchymal stem cell-based therapy for PD rodent models were identified from PubMed, EMBASE, and Web of Science through September 30, 2020 using the following search strategy: (“mesenchymal stem cells” OR “mesenchymal stromal cells” OR “mesenchymal stem cell” OR “mesenchymal stromal cell” OR “bone marrow stem cell” OR “bone marrow-derived stromal cell”) AND (“Parkinson's disease” OR “Parkinson disease” OR “PD”). The publication language was limited to English. We also searched the reference lists of eligible studies.

### Inclusion and Exclusion Criteria

The studies' eligibility criteria were set up according to the PICOS-scheme (population, intervention, control, outcome and study design) (Riva et al., 2012). The inclusion criteria were as follows: (i) Parkinson's animal model (rodent models); (ii) testing the effects of unmodified BMSCs in at least one experimental group; (iii) setting sham-controlled group or condition; (iv) providing adequate data on behavioral testing or densitometry of tyrosine hydroxylase-positive (TH^+^) staining in the SNpc; (v) study: experimental studies presented in original research articles; and (vi) published in English. The exclusion criteria were as follows: (i) researches that only evaluated the efficacy of transfected or modified cell transplantation; (ii) studies that only tested stem cells other than bone marrow mesenchymal stem cells; (iii) BMSCs administered before PD model.

### Study Selection

After removal of duplicates, all published articles were conducted by two investigators independently. When the two investigators agreed, irrelevant studies were excluded. All relevant articles were retrieved for a comprehensive review, and the two researchers independently evaluated these articles using criteria outlined above. Any differences or uncertainties were resolved through consensus and judged by a third investigator when necessary.

### Data Abstraction

The following information were abstracted by two investigators independently and entered electronically: authors, year published, study country, source of BMSCs, species of animals, animal model, animal gender, anesthetic type, cell dose, delivery route, and timing of BMSCs, follow-up (the longest observation time of outcomes after BMSCs administration), the outcomes data.

When only graphs were available, values were obtained from images using GetData Graph Digitizer software. The average reading of the two researchers were used to analysis data. If the standard deviation was not reported, standard error was converted to standard deviation by multiplying the square root of the group size. If a research contained multiple experimental groups differentiated by cell dose or delivery route and timing that were contrasted with the control group, these experimental groups would be included separately as independent studies. If the outcomes were evaluated at different follow-up times, only the longest one was extracted.

### Quality Assessment

To evaluate the quality of the eligible studies, we used the Collaborative Approach to Meta-Analysis and Review of Animal Data from Experimental Studies (CAMARADES) checklists (Macleod et al., 2004), which consist of the following items: (1) publication in a peer-reviewed journal, (2) statements describing temperature control, (3) randomized treatment allocation, (4) allocation concealment, (5) use of aged animal models, (6) blind assessment of outcome, (7) avoidance of anesthetics with significant intrinsic neuroprotective activity, such as ketamine, (8) reporting of a sample size calculation, (9) statement of compliance with regulatory requirements, and (10) declarations of potential conflicts of interest. A sum of the quality scores was recorded for each study, with a total score of 10 points. Two researchers independently scored the studies. Any differences or uncertainty were resolved by consensus.

### Statistical Analysis

Combined effect size was calculated as standardized mean difference (SMD) between BMSCs treated group and control group. The random-effects model and Hedges calculation (Durlak, 2009) were applied to get the pooled effect size, and all analysis was performed with Stata software (version 12.1). Overall, an effect size of 0.2 represents a small effect, 0.5 and 0.8 represent medium and large, respectively (Schulz et al., 1995). A *P*-value < 0.05 was considered statistically significant. The *I*^2^ statistic was used to analyze heterogeneity, and it was defined as low (25–50%), moderate (50–75%), or high (>75%) (Higgins et al., 2003).

Seven clinical characteristics were used to grouping the effect size of outcome: BMSCs species (Allogeneic or Xenogeneic); BMSCs dose (≤ 1E5, 1E5–1E6, ≥1E6); delivery time (< 14, 14–21, ≥21 days); follow-up duration (≤ 4, 4–8, >8 weeks); delivery route (intrastriatal, intravenous, intracerebral, intranigral, intranasal); PD model [6-hydroxydopamine (6-OHDA), 1-methyl-4-phenyl-1,2,3,6-tetrahydropyridine (MPTP), Roteneone]; Gender (male, female). Pre-specified subgroup analysis and meta-regression analysis (Higgins and Thompson, 2002) were performed to study the possible relations between the outcomes and the above clinical characteristics.

Publication bias was evaluated using funnel plots (Sterne et al., 2011), and the symmetry of funnel plots was performed with Egger regression (Egger et al., 1997). If necessary, any non-negligible bias would be corrected using the trim-and-fill approach (Sterne et al., 2001).

## Results

### Study Inclusion

**Figure 1:**
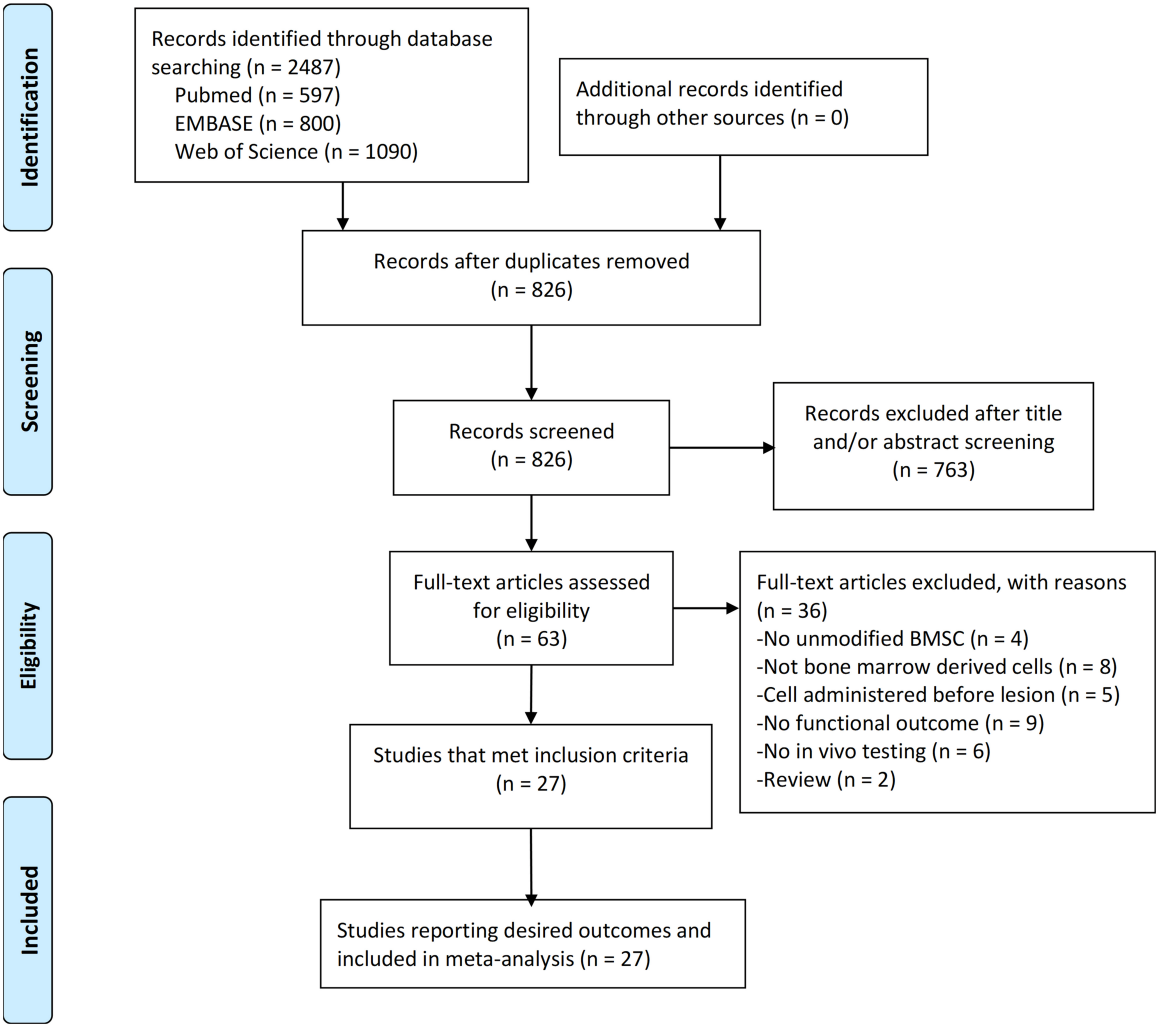
PRISMA flow diagram for review and selection process of studies included in meta-analysis of BMSCs in rodent models of Parkinson's Disease.

Electronic searching identified 597 articles in PubMed, 800 articles in EMBASE, and 1,090 articles in Web of Science. After removal of duplicates, 826 articles were screened by abstract and/or title, resulting in 763 articles excluded. By reading the full text of the remaining 63 articles, 36 were excluded due to review, not having unmodified BMSC experiments, not bone marrow derived cells, no *in vivo* experiment, BMSCs administered before lesion, or no primary outcomes. Therefore, 27 studies were included in the meta-analysis (Figure 1). All studies published in peer-reviewed journals.

### Study Characteristics

The characteristics of the 27 studies are summarized in Supplementary Tables 1, 2. All studies were carried out in rodents (rats and mice). Intervention included MSCs obtained from mice, rat, or human bone marrow. The most common PD model was the 6-OHDA, although other methods were also used, such as the Rotenone and MPTP. Following induction of PD, BMSCs were administrated either immediately or over a period varying from 24 h to 5 weeks. The mean follow-up ranged from 8 days to 20 weeks. The most common delivery route used for BMSCs was intrastriatal route. Others used were the intravenous, intracarotid, intracerebral (into left/right ventricle), intrathecal (into subarachnoid space), and intranasal route. Histopathological outcome was assessed by densitometry of TH^+^ staining in 18 studies. Behavioral outcomes were evaluated by rotational behavior in 20 researches, rotarod test in five researches, open field test in three researches, limb function (cylinder, adjusting step, staircase tests, treadmill locomotion test, and paw-reaching tests) in eight studies, and forced swimming test in one study. Considering that densitometry of TH^+^ staining in SNpc and rotational behavior are the most common evaluations used in rodent studies of PD, we took them as co-primary outcomes in this review.

### Quality Assessment

The quality assessment of included studies is summarized in Table 1, the details are presented in Supplementary Table 3. The quality scores varied from 2 to 8, with a mean value of 4.7. According to our statistical results, all the articles were published in a peer-reviewed journal and claimed compliance with animal welfare regulations. No study used aged animals.

**Table 1:** Percentage of included studies satisfying each criterion of CAMARADES checklists.

**Quality score criterion**	**Percentage of qualified studies (%)**
Publication in a peer-reviewed journal	100
Control of temperature	44
Randomized treatment allocation	51.8
Allocation concealment	29.6
Use of aged animal models	0
Blind assessment of outcome	29.6
Avoidance neuroprotective anesthetics	55.5
Sample size calculation	0
Compliance with animal welfare regulations	100
Statement of conflict of interest	63

### Meta-Analysis

**Figure 2:**
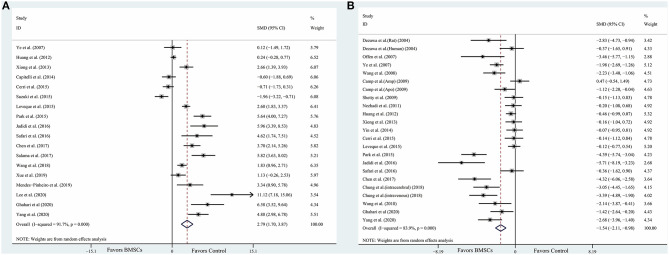
Forest plot shows mean effect size and 95% CI for **(A)** densitometry of TH^+^ staining in the substantia nigra pars compacta, **(B)** rotational behavior between BMSCs therapy group and control group in individual trials and all studies combined. Weights have been calculated using random effects model. Degree of heterogeneity in the pooled estimates is represented at I^2^ statistic. SMD, standardized mean difference; BMSCs, Bone marrow stromal cells.

The data were extracted from the studies included (Supplementary Table 2). The composite weighted mean (95% confidence interval) effect size for densitometry of TH^+^ staining was 2.79 (1.70, 3.87) (*P* < 0.001) (Figure 2A); for rotational behavior was −1.54 (−2.11, −0.98) (*P* < 0.001) (Figure 2B). We also conducted pooled analysis for rotarod tests (*n* = 6) and limb function (*n* = 7). The result was similar: The composite weighted mean (95% CI) effect size for rotarod tests was 1.77 (0.40, 3.15) (*P* < 0.001, *I*^2^ = 85.6%), and 0.28 (−0.52, 1.08) (*P* = 0.001, *I*^2^ = 73.4%) for limb function. The Higgins *I*^2^ index indicated significant heterogeneity among four outcomes (*P* < 0.001).

### Stratified Analysis and Meta-Regression Analysis

Table 2 summarizes the data of primary outcomes in diverse subgroup analysis. In general, significant efficacy of BMSCs transplantation were observed in most subgroups. Partial subgroups fail to reach the statistical significance (*P* < 0.05), which may be caused by insufficient sample size. Although significant difference was found between groups in partial subgroup analysis, we could not find the source of heterogeneity. In the most subgroups with two or more studies included, substantial heterogeneity was found (*I*^2^ > 75%). In order to further investigate the unexplained heterogeneity, multivariate meta-regression was used to test the influence of all clinical characteristics on the outcomes. However, for densitometry of TH^+^ staining, no significant sources of heterogeneity were found. For rotational behavior, the administration time was the significant source of heterogeneity (*P* = 0.009).

**Table 2:** Subgroup analysis of rotational behavior and densitometry of TH^+^ staining in animal models of Parkinson's disease associated with BMSC therapy.

**Variable**	**Densitometry of TH**^****+****^ **staining in the SNpc**						**Rotation behavior**					
	**No. of reports**	**Pooled estimates (95% CI)**	**Q statistic**	***P*-value for heterogeneity**	***I^**2**^* value (%)**	**Between group *P*-value**	**No. of reports**	**Pooled estimates (95% CI)**	**Q statistic**	***P*-value for heterogeneity**	***I^**2**^* value (%)**	**Between group *P*-value**
BMSC species						<0.001						0.013
Allogeneic (mice/rats)	14	2.14 (1.03, 3.26)	146.17	< 0.001	91.10%		16	−1.35 (−1.97, −0.73)	80.95	<0.001	81.50%	
Xenogeneic (human)	4	5.22 (2.54, 7.90)	20.89	<0.001	85.60%		7	−1.91 (−3.19, −0.63)	49.3	<0.001	87.80%	
BMSCs dose						<0.001						0.125
≤ 1E5	4	0.72 (−1.10, 2.53)	32.12	<0.001	90.70%		4	−1.84 (−3.54, −0.15)	22.07	<0.001	86.40%	
1E5–1E6	6	3.42 (1.58, 5.27)	28.94	<0.001	82.70%		9	−1.68 (−2.56, −0.79)	58.68	<0.001	83.00%	
≥1E6	8	3.59 (1.65, 5.52)	103.03	<0.001	93.20%		7	−1.20 (−2.23, 0.16)	35.33	<0.001	87.30%	
Administration time						0.101						<0.001
<2 weeks	5	3.31 (0.56, 6.06)	52.29	<0.001	90.00%		4	−0.58 (−1.45, 0.29)	7.06	0.07	57.50%	
2–3 weeks	5	1.82 (0.18, 3.47)	59.64	<0.001	93.30%		7	−0.94 (−1.82, −0.06)	37.69	<0.001	84.10%	
≥3 weeks	4	1.22 (−1.63, 4.07)	36.35	<0.001	91.70%		5	−3.03 (−3.96, −2.11)	8.68	0.07	53.90%	
Follow-up period						<0.001						0.026
≤ 4 weeks	10	3.18 (1.55, 4.81)	115.75	<0.001	92.20%		11	−1.78 (−2.78, −0.78)	77.37	<0.001	87.10%	
4–8 weeks	6	3.10 (1.23, 4.97)	50.37	<0.001	90.10%		8	−1.14 (−1.95, −0.34)	38.14	<0.001	81.60%	
>8 weeks	2	−0.99 (−3.02, 1.05)	4	0.045	75%		4	−1.87 (−3.23, −0.51)	13.59	0.004	77.90%	
Administration route						<0.001						<0.001
Intrastriatal	9	1.06 (0.01, 2.12)	59.32	<0.001	86.50%		14	−1.13 (−1.73, −0.53)	60.19	<0.001	78.40%	
Intravenous	4	4.65 (2.52, 6.77)	16.21	0.001	81.50%		5	−1.88 (−3.53, −0.24)	42.24	<0.001	90.50%	
Intracerebral	2	5.26 (3.73, 6.79)	0.44	0.507	0		3	−3.47 (−4.90, −2.04)	4.62	0.099	56.70%	
Intranasal	1	5.82 (3.63, 8.02)	NA	NA	NA		0	NA	NA	NA	NA	
Intracarotid	1	−0.71 (−1.73, 0.31)	NA	NA	NA		1	−0.14 (−1.12, 0.84)	NA	NA	NA	
Intrathecal	1	11.12 (7.18, 15.06)	NA	NA	NA		0	NA	NA	NA	NA	
PD model						0.001						0.006
6-OHDA	13	2.86 (1.54, 4.17)	162.80	<0.001	92.60%		21	−1.57 (−2.17, −0.97)	126.25	<0.001	84.20%	
Roteneone	2	4.11 (1.02, 7.20)	5.98	0.014	83.30%		1	−0.16 (−1.04, −0.72)	NA	NA	NA	
MPTP	3	1.73 (−1.14, 4.60)	21.88	<0.001	68.80%		1	−2.68 (−3.96, −1.40)	NA	NA	NA	
Gender						0.004						0.736
Male	15	3.15 (1.94, 4.36)	155.07	<0.001	91.60%		18	−1.74 (−2.43, −1.05)	113.31	<0.001	85.00%	
Female	3	0.27 (−2.56, 3.11)	25.78	<0.001	92.20%		4	−0.72 (−1.85, 0.41)	18.6	<0.001	83.90%	

*BMSCs, bone marrow stromal cells; PD, Parkinson's disease; TH^+^, tyrosine hydroxylase positive; NA, not available; SNpc, substantia nigra pars compacta*.

### Publication Bias

**Figure 3:**
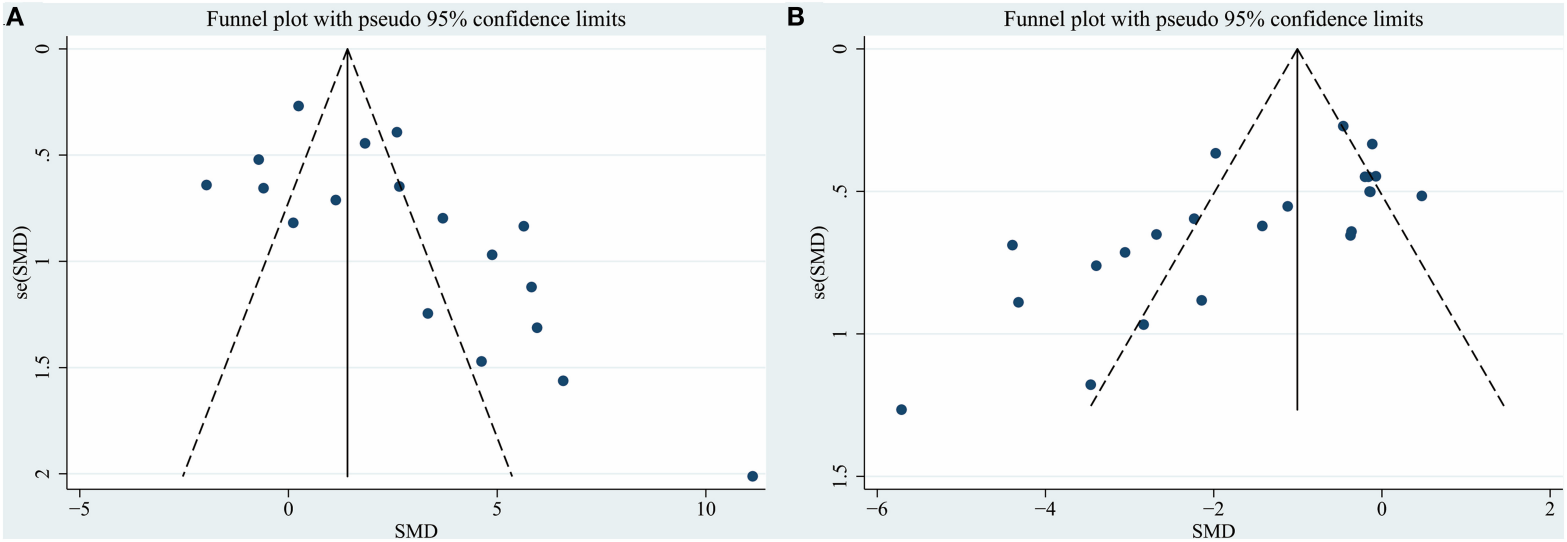
Funnel plot for **(A)** densitometry of TH^+^ staining in the substantia nigra pars compacta, **(B)** rotational behavior. Each dot in the figure represents a study, with the y-axis signifying study quality and the x-axis showing the study results. SMD, standardized mean difference.

We assessed publication bias by funnel plots (Figure 3A for densitometry of TH^+^ staining; Figure 3B for rotational behavior). No evident publication bias was observed by visual inspection. The Egger test presented significant publication bias for rotational behavior (*P* = 0.001) but not for densitometry of TH^+^ staining (*P* = 0.061). After adopting trim-and-fill correction for rotational behavior outcomes, the estimated value remained unchanged.

## Discussion

**Figure 4:**
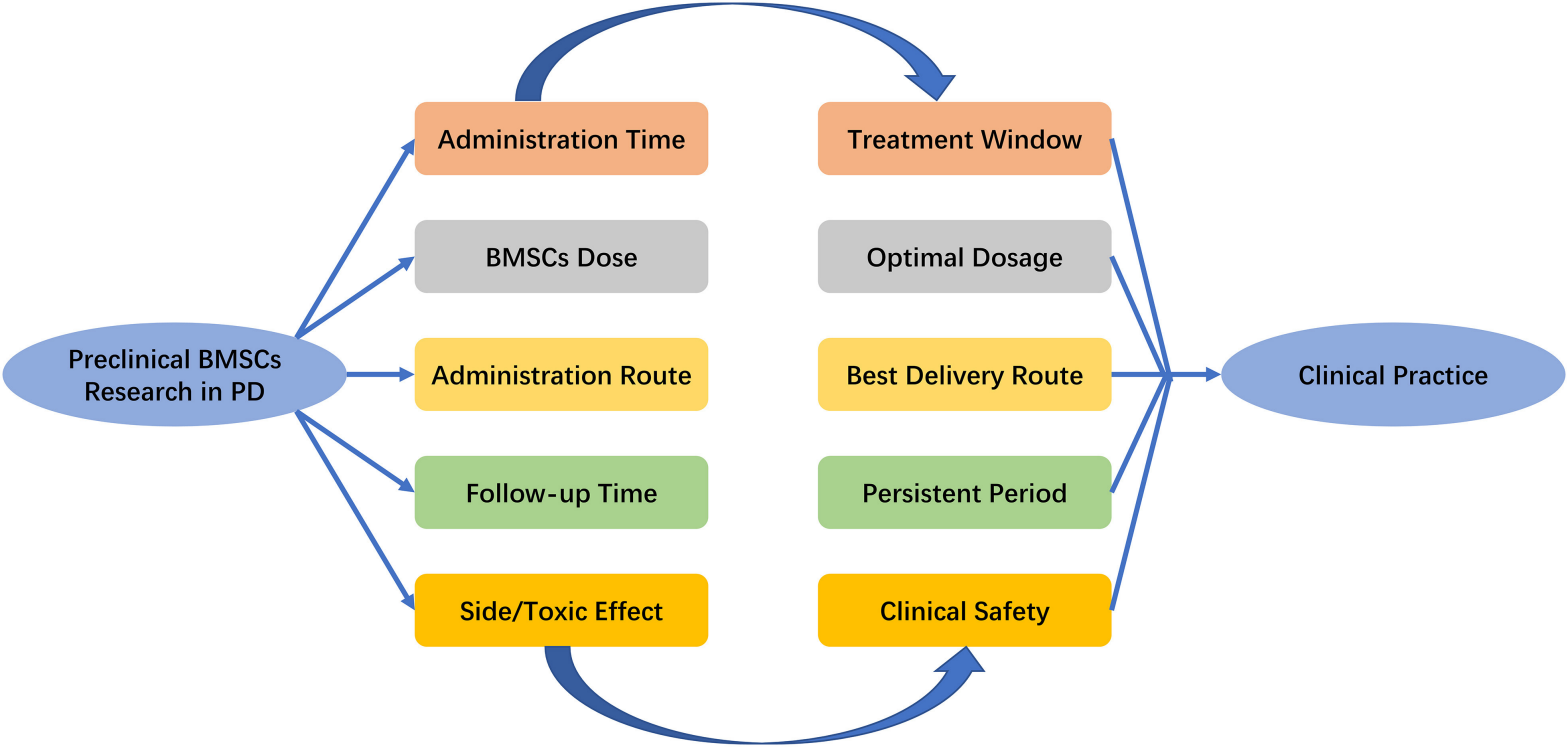
Key issues that the settlement of which would facilitate the transition of BMSCs research in PD from bench to bedside. PD, Parkinson's Disease; BMSCs, Bone marrow stromal cells.

The first open-labeled clinical study ascertaining the safety and efficacy of BMSCs in PD patients was performed in 2010, where seven patients who received unilateral injections of autologous BMSCs into the sublateral ventricular zone demonstrated the safety of transplantation (Venkataramana et al., 2010). The pilot clinical study in 2012 demonstrated a modest improvement during “on” and “off” period on the UPDRS scoring system in early-stage PD patients (Venkataramana et al., 2012). Table 3 includes the related clinical trials that are registered with ClinicalTrails.gov. Although BMSCs have shown a promising role in PD in initial clinical pilot studies, there are a number of uncertain questions with respect to the delivery timing, route of administration, and BMSCs dose. As the evidence generated from animal model provide a framework for designing clinical trials, it is important to explore the pooled effects of preclinical studies. What stages (acute/chronic) of PD are indicated for stem cell therapy, what doses of BMSCs are optimal, how do we deliver BMSCs, how long to survive within the hostile ischemic microenvironment, and how do we improve neurological function? (Figure 4). Prior meta-analysis either took a broader approach to multiple different mesenchymal stem cell type (Riecke et al., 2015) or only included the induced pluripotent stem cell (Zhang Y. et al., 2018). These studies did not generate an effect size of histological outcomes of PD, only evaluate the behavioral outcomes. TH^+^ staining has been frequently examined as histological identification of dopaminergic neurons (Barzilay et al., 2008). Thus, we took densitometry of TH^+^ staining as the measure of histological outcomes in PD animal models.

**Table 3:** Clinical trials using BMSCs in PD that are registered with ClincalTrials.gov (as of October 2020).

**No. NCT**	**Years**	**Type of trial**	**Locations**	**Recruitment status**	**Phase**	**Ages (years)**	**Allo/Auto**	**Route of admistration**	**No. of BMSCs**	**Follow-up period**
NCT00976430	2009	Open Label	India	Terminated	Not Applicable	35–70	Autologous	Stereotactically (striatum)	Not Applicable	18 months
NCT01446614	2011	Open Label	China	Unknown	Phase 1/2	30–65	Autologous	Intravenous	6 × 10^5^ per kg, qw, for 4 weeks	12 months
NCT02611167	2015	Open Label	United States	Completed	Phase 1	45–70	Allogeneic	Intravenous	1/3/6/10 × 10^6^ per kg	52 weeks
NCT04506073	2020	Randomized controlled trial	United States	Not yet recruiting	Phase 2	50–79	Allogeneic	Intravenous	3 infusions of 10 × 10^6^ per kg 3 months	78 weeks

*BMSCs, bone marrow stromal cells*.

### Main Findings

This meta-analysis suggests the following: (1) Based on the defined quantification of the absolute value of the effect, we observed effect sizes of BMSCs on histological and behavioral outcomes were very large. All estimates other than limb function were statistically significant. In general, this meta-analysis suggested that deficits of both motor function and TH^+^ levels in rodent models were alleviated by BMSCs therapy. (2) Administration time was correlated with effect size in densitometry of TH^+^ staining and rotational behavior. BMSCs therapy initiated more than 3 weeks post-PD showed the greatest efficacy, followed by 2–3 weeks post-PD, and then therapy initiated within 2 weeks. (3) Intravenous seems to be more effective compared with intrastriatal to improve rotational behavior and increase densitometry of TH^+^ staining. But the number of animals included for intravenous administration route in the pooled analysis were small, this comparison needs to be proved by more studies. (4) Xenogenic and allogenic cells showed similar beneficial effects for rotation behavior, although the latter increased the densitometry of TH^+^ staining to a larger extent. But the number of studies included for xenogeneic group were small. (5) The included studies do not clearly provide a specific effective dose of BMSCs. With higher dose levels (>1 × 10^5^), a greater effect size of BMSCs therapy on densitometry of TH^+^ staining was observed. Generally, the above various subgroup analysis can only generate hypothesis rather than confirming them.

### Possible Mechanism of Neuroprotective Effects

**Figure 5:**
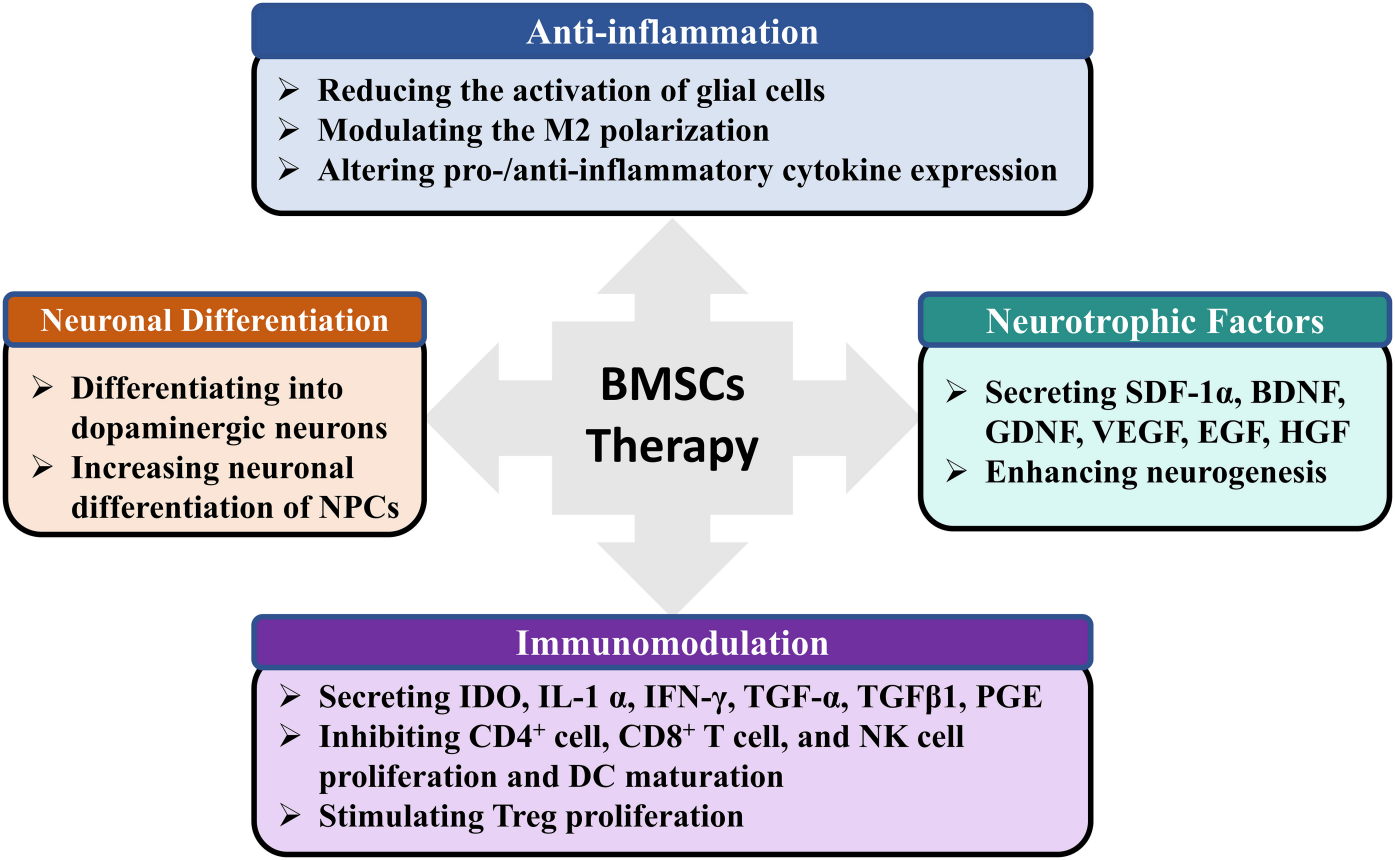
The possible mechanisms of BMSCs therapy for PD. PD, Parkinson's Disease; BMSCs, Bone marrow stromal cells; GDNF, SDF-1α, Stromal cell-derived factor 1a; BDNF, Brain-derived neurotrophic factor; Glial cell-derived neurotrophic factor; VEGF, Vascular endothelial growth factor; EGF, Epidermal growth factor; HGF, Hepatocyte growth factor; NPCs, Neural progenitor cells; IDO, Indoleamine-2,3-dioxygenase; IFNγ, Interferon γ; TGF-α, Transforming growth factor alpha; TGFβ1, Transforming growth factor beta 1; PGE, Prostaglandin; DC, Dendritic cell.

MSCs-based therapy is a multimodal treatment for nervous system diseases (Badyra et al., 2020). The precise mechanism by which BMSCs may exert beneficial effects in PD is still being elucidated, but it appears that multiple mechanisms may contribute (Glavaski-Joksimovic and Bohn, 2013; Fan et al., 2020). First, anti-inflammatory properties of BMSCs. BMSCs administration in rats dramatically decreased dopaminergic neuronal loss in the SNpc, which was obviously accompanied by reduced activation of microglia (Park et al., 2008; Suzuki et al., 2015), as well as the expression of inducible nitric oxide synthase and tumor necrosis factor-alpha (Kim et al., 2009). By inducing M2 microglia polarization, BMSCs can enhance α-synuclein clearance *in vitro* model (Park et al., 2016). Activated by inflammatory signals, MSCs also secrete the anti-inflammatory protein TNF-α-stimulated gene 6 protein (Choi et al., 2011), IL-6, and IL-10 (Jinfeng et al., 2016). But whether BMSCs can promote the switch of M1 microglia status into M2 phenotype and increase the anti-inflammatory protein in PD animal model needs further study. Second, paracrine activity of BMSCs. BMSCs can secrete factors including brain-derived neurotrophic factor, glial cell-derived neurotrophic factor, and vascular endothelial growth factor (Sadan et al., 2009), which can improve neuronal survival. Glial cell-derived neurotrophic factor released by BMSCs may protect catecholaminergic and serotonergic neuronal perikarya and transporter function (Whone et al., 2012), which may be effective for abrogating the non-motor symptoms of PD. As an important medium for paracrine effects, BMSCs-derived exosomes contain a variety of biomolecules, such as proteins, messenger RNA, and miRNA (e.g., miR-124, and miR-145), which contribute to neuroprotection and mediate immunomodulatory effects (Kojima et al., 2018; Mendes-Pinheiro et al., 2019). Finally, neuronal differentiation of BMSCs. Previous report has presented that BMSCs could be induced to form dopamine decarboxylase-positive cells along the line of restoring/replacing dopamine cell loss (Jiang et al., 2002). Alleviating motor symptoms of induced BMSCs transplantation has been tested in animal model of PD (Dezawa et al., 2004). Whether BMSCs can differentiate to a variety of neuronal phenotypes (such as noradrenergic, serotonergic, and cholinergic cell types), and improve the non-motor symptoms of PD requires further research. The above mechanisms have shown that transplantation of BMSCs can not only improve PD symptom directly by neuronal differentiation, but also trigger endogenous brain repair through the modulation of anti-inflammatory cytokines, proteomes, and neurotrophic factors (Figure 5).

### Limitations

There are several limitations to our meta-analysis. First, our approach can only include the studies that have already been published in English. Unpublished data may change our results. In addition, none of the included studies investigated the safety of BMSCs injection on PD in animal models. we are incapable of evaluating the clinical safety of BMSCs application. Finally, a good study should have an adequate sample size with a formal calculation (Campbell et al., 1995). Nevertheless, no studies in the meta-analysis conducted sample calculation, which indicated the lack of statistical power to ensure proper estimation of the treatment effects (Schulz and Grimes, 2005).

### Future Direction

There is significant work to be done for the future clinical translation. Firstly, most research subjects are rats, and their similarities to humans are limited. Therefore, since the outcomes for rodent model cannot be directly extended to humans, primates should be used to obtain more results. Secondly, PD is an age-related disease, and the majority of PD patients are elderly. As previously stated, data shows a direct relation between age and the occurrence of PD (Tysnes and Storstein, 2017). Thus, the impact of age should be considered in preclinical studies given that the epidemiology of PD and response to therapy may vary widely in the developing, juvenile, adult, and elderly brains. However, the current research models are based almost exclusively on healthy adult animals. It is doubtful whether cell therapy can achieve the same treatment effect in elderly Parkinson's animal model.

## Conclusion

Preclinical researches have showed the potential role of BMSCs to be an effective therapy for PD patients. But, determining the clinical parameters by our meta-analysis is inevitably confounded by high publication bias. Considering the limited internal and external validity, our conclusions should be confirmed in more strictly randomized control studies and carefully interpreted in relation to the design of future animal studies or clinical translation. Generally, the use of BMSCs as a novel therapeutic strategy for PD is promising.

## Data Availability Statement

All datasets generated for this study are included in the article/Supplementary Material.

## Author Contributions

JL analyzed the data. JH and YH supervised the project. JL and ZH wrote the paper. All authors contributed to the article and approved the submitted version.

## Conflict of Interest

The authors declare that the research was conducted in the absence of any commercial or financial relationships that could be construed as a potential conflict of interest.
