# Supplementary material for: Effect of Bone Marrow Stromal Cells in Parkinson's Disease Rodent Model: A Meta-Analysis
Source: Front Aging Neurosci. 2020 Dec 11;12:539933. doi: 10.3389/fnagi.2020.539933 (PMC7759665; doi:10.3389/fnagi.2020.539933)
Supplement: Supplementary file 1 [file Table_1.pdf]

## Supplementary Tables

**Supplementary Table 1. Characteristics of the studies included for the meta-analysis.** BMSCs- Bone marrow stromal cells; 6-OHDA- 6-hydroxydopamine; MPTP- 1-methyl-4-phenyl-1,2,3,6-tetrahydropyridine; ip- intraperitoneally; NA- not available

| Sl.no. | Reference | Country | Intervention | Species, gender | Model, anesthetic type | Dose (MSC) | Time of administration | Follow-up (weeks) | Route of administration |
|---|---|---|---|---|---|---|---|---|---|
| **1** | (Li et al., 2001) | USA | Mice BMSCs | Mice, male | MPTP, inhalation | $3x10^5$ | 7 days | 4 weeks | Intravenous |
| **2** | (Dezawa et al., 2004) | Japan | Rat/Human BMSCs | Rats, male | 6-OHDA, ip | $1x10^5$ | NA | 10 weeks | Intrastriatal |
| **3** | (Offen et al., 2007) | Israel | Mice BMSCs | Mice, male | 6-OHDA, ip | $2x10^5$ | 21 days | 12 weeks | Intrastriatal |
| **4** | (Ye et al., 2007) | China | Rat BMSCs | Rats, female | 6-OHDA, ip | $6x10^5$ | 30 days | 20 weeks | Intrastriatal |
| **5** | (Wang et al., 2008) | China | Mice BMSCs | Rats | 6-OHDA, ip | NA | NA | 6 weeks | Intrastriatal |
| **6** | (Camp et al., 2009) | USA | Rat BMSCs | Rats, female | 6-OHDA, ip | $2x10^5$ | Immediately | 16-20 days | Intrastriatal |
| **7** | (Shetty et al., 2009) | India | Human BMSCs | Rats, male | 6-OHDA, ip | $2\text{-}3x10^5$ | 4-6 weeks | 12 weeks | Intrastriatal |
| **8** | (Nezhadi et al., 2011) | Iran | Rat BMSCs | Rats, male | 6-OHDA, ip | $2x10^5$ | 16 days | 8 weeks | Intrastriatal |
| **9** | (Huang et al., 2012) | China | Rat BMSCs | Rats, male | 6-OHDA, ip | $5x10^3$ | 14 days | 8 weeks | Intrastriatal |
| **10** | (Xiong et al., 2013) | China | Human BMSCs | Rats, female | Rotenone, ip | $1x10^6$ | 14 days | 8 weeks | Intrastriatal |
| **11** | (Capitelli et al., 2014) | Brazil | Rat BMSCs | Rats, male | MPTP, ip | $1/5x10^6$ | Immediately/ 1 day | 8 days | Intravenous |
| **12** | (Yin et al., 2014) | China | Rat BMSCs | Rats, male | 6-OHDA, ip | $1x10^6$ | 14 days | 8 weeks | Intrastriatal |
| **13** | (Cerri et al., 2015) | Italy | Rat BMSCs | Rats, male | 6-OHDA, ip | $1x10^6$ | 14 days | 4 weeks | Intracarotid |

| | | | | | | | | | |
|---|---|---|---|---|---|---|---|---|---|
| **14** | (Leveque et al., 2015) | Korea | Rat BMSCs | Rats, male | 6-OHDA, intramuscular | $2x10^6$ | NA | 4 weeks | Intravenous |
| **15** | (Park et al., 2015) | Japan | Human BMSCs | Rats, male | 6-OHDA, ip | $1x10^7$ | 16 days | 4 weeks | Intravenous |
| **16** | (Suzuki et al., 2015) | France | Rat BMSCs | Rats, female | 6-OHDA, ip | $1x10^5$ | 30 days | 16 weeks | Intrastriatal |
| **17** | (Jadidi et al., 2016) | Iran | Rat BMSCs | Rats, male | 6-OHDA | $2x10^5$ | NA | 2 weeks | Intracerebral (Left ventricle) |
| **18** | (Safari et al., 2016) | Iran | Rat BMSCs | Rats, male | 6-OHDA, ip | $2x10^5$ | 7 days | 5 weeks | Intravenous |
| **19** | (Chen et al., 2017) | China | Rat BMSCs | Rats, male | 6-OHDA, ip | $6x10^4$ | 21 days | 8 weeks | Intrastriatal |
| **20** | (Salama et al., 2017) | Malaysia | Mice BMSCs | Mice | Rotenone, ip | $5x10^5$ | Immediately | 10 days | Intranasal |
| **21** | (Chung et al., 2018) | China | Human BMSCs | Mice, male | 6-OHDA, inhalation | $3.6x10^5$ | 21 days | 2 weeks | Intracerebral (Right ventricle)/ Intravenous |
| **22** | (Wang et al., 2018) | China | Rat BMSCs | Rats, male | 6-OHDA, ip | $2\text{-}3x10^5$ | 14 days | 4 weeks | Intrastriatal |
| **23** | (Xue et al., 2019) | China | Mice BMSCs | Mice, male | MPTP, ip | $1x10^4$ | 1 day | 4 weeks | Intrastriatal |
| **24** | (Mendes-Pinheiro et al., 2019) | Portugal | Human BMSCs | Rats, male | 6-OHDA, ip | $2x10^5$ | 35 days | 7 weeks | Intrastriatal |
| **25** | (Lee et al., 2020) | Korea | Human BMSCs | Rats, male | 6-OHDA, ip | $2x10^7$ | 14 days | 4 weeks | Intrathecal (Subarachnoid space) |
| **26** | (Ghahari et al., 2020) | Iran | Rat BMSCs | Rats, male | 6-OHDA, ip | $2x10^6$ | 7 days | 4 weeks | Intravenous |
| **27** | (Yang et al., 2020) | China | Mice BMSCs | Mice, male | MPTP, NA | $1x10^{12}$ | NA | 8 weeks | Intracerebral |

**Supplementary Table 2. Outcomes of the studies included for the meta-analysis.** BMSCs- Bone marrow stromal cells; TH- Tyrosine hydroxylase; SNpc- substantia nigra pars compacta

| Sl.no. | Author | Year | Intervention | Densitometry of $TH^+$ staining in the SNpc | Rotational behavior | limb function | Rotarod test | Open field test | Forced swimming test |
|---|---|---|---|---|---|---|---|---|---|
| **1** | Li et al. | 2001 | Mice BMSCs | | | | ✓ | | |
| **2** | Dezawa et al. | 2004 | Rat/Human BMSCs | | ✓ | ✓ | | | |
| **3** | Offen et al. | 2007 | Mice BMSCs | | ✓ | | | | |
| **4** | Ye et al. | 2007 | Rat BMSCs | ✓ | ✓ | | | | |
| **5** | Wang et al. | 2008 | Mice BMSCs | | ✓ | | | | |
| **6** | Camp et al. | 2009 | Rat BMSCs | | ✓ | ✓ | ✓ | | |
| **7** | Shetty et al. | 2009 | Human BMSCs | | ✓ | | | | |
| **8** | Nezhadi et al. | 2011 | Rat BMSCs | | ✓ | | | | |
| **9** | Huang et al. | 2012 | Rat BMSCs | ✓ | ✓ | | | | |
| **10** | Xiong et al. | 2013 | Human BMSCs | ✓ | ✓ | | | | |
| **11** | Capitelli et al. | 2014 | Rat BMSCs | ✓ | | | | ✓ | ✓ |
| **12** | Yin et al. | 2014 | Rat BMSCs | | ✓ | | | | |
| **13** | Cerri et al. | 2015 | Rat BMSCs | ✓ | ✓ | ✓ | | | |
| **14** | Leveque et al. | 2015 | Rat BMSCs | ✓ | ✓ | | | | |

| | | | | | | | | |
|---|---|---|---|---|---|---|---|---|
| **15** | Park et al. | 2015 | Human BMSCs | ✓ | ✓ | | | |
| **16** | Suzuki et al. | 2015 | Rat BMSCs | ✓ | | ✓ | | |
| **17** | Jadidi et al. | 2016 | Rat BMSCs | ✓ | ✓ | | | |
| **18** | Safari et al. | 2016 | Rat BMSCs | ✓ | ✓ | | | |
| **19** | Chen et al. | 2017 | Rat BMSCs | ✓ | ✓ | | | |
| **20** | Salama et al. | 2017 | Mice BMSCs | ✓ | | | | ✓ |
| **21** | Chung et al. | 2018 | Human BMSCs | | ✓ | | ✓ | |
| **22** | Wang et al. | 2018 | Rat BMSCs | ✓ | ✓ | | | |
| **23** | Xue et al. | 2019 | Mice BMSCs | ✓ | | | ✓ | ✓ |
| **24** | Mendes-Pinheiro et al. | 2019 | Human BMSCs | ✓ | | ✓ | ✓ | |
| **25** | Lee et al. | 2020 | Human BMSCs | ✓ | | ✓ | | |
| **26** | Ghahari et al. | 2020 | Rat BMSCs | ✓ | ✓ | | | |
| **27** | Yang et al. | 2020 | Mice BMSCs | ✓ | ✓ | ✓ | | |

**Supplementary Table 3. Quality scores of the studies included for the meta-analysis.**

| Sl.no. | Author | Year | 1 | 2 | 3 | 4 | 5 | 6 | 7 | 8 | 9 | 10 | Quality score |
|---|---|---|---|---|---|---|---|---|---|---|---|---|---|
| **1** | Li et al. | 2001 | 1 | 0 | 0 | 0 | 0 | 0 | 0 | 0 | 1 | 0 | **2** |
| **2** | Dezawa et al. | 2004 | 1 | 0 | 0 | 0 | 0 | 0 | 1 | 0 | 1 | 1 | **4** |
| **3** | Offen et al. | 2007 | 1 | 1 | 0 | 0 | 0 | 0 | 1 | 0 | 1 | 0 | **4** |
| **4** | Ye et al. | 2007 | 1 | 0 | 1 | 0 | 0 | 0 | 1 | 0 | 1 | 0 | **4** |
| **5** | Wang et al. | 2008 | 1 | 0 | 0 | 0 | 0 | 0 | 1 | 0 | 1 | 0 | **3** |
| **6** | Camp et al. | 2009 | 1 | 0 | 0 | 1 | 0 | 1 | 1 | 0 | 1 | 1 | **6** |
| **7** | Shetty et al. | 2009 | 1 | 0 | 1 | 1 | 0 | 0 | 0 | 0 | 1 | 0 | **4** |
| **8** | Nezhadi et al. | 2011 | 1 | 0 | 1 | 0 | 0 | 0 | 0 | 0 | 1 | 0 | **3** |
| **9** | Huang et al. | 2012 | 1 | 0 | 1 | 0 | 0 | 0 | 0 | 0 | 1 | 1 | **4** |
| **10** | Xiong et al. | 2013 | 1 | 1 | 1 | 1 | 0 | 1 | 1 | 0 | 1 | 1 | **8** |
| **11** | Capitelli et al. | 2014 | 1 | 1 | 1 | 0 | 0 | 0 | 1 | 0 | 1 | 1 | **6** |
| **12** | Yin et al. | 2014 | 1 | 0 | 0 | 0 | 0 | 0 | 1 | 0 | 1 | 1 | **4** |
| **13** | Cerri et al. | 2015 | 1 | 1 | 1 | 0 | 0 | 0 | 1 | 0 | 1 | 1 | **6** |
| **14** | Leveque et al. | 2015 | 1 | 1 | 0 | 0 | 0 | 0 | 1 | 0 | 1 | 0 | **4** |
| **15** | Park et al. | 2015 | 1 | 1 | 0 | 0 | 0 | 0 | 0 | 0 | 1 | 0 | **3** |
| **16** | Suzuki et al. | 2015 | 1 | 0 | 0 | 0 | 0 | 0 | 0 | 0 | 1 | 1 | **3** |

| | | | | | | | | | | | | | |
|---|---|---|---|---|---|---|---|---|---|---|---|---|---|
| **17** | Jadidi et al. | 2016 | 1 | 1 | 1 | 1 | 0 | 0 | 0 | 0 | 1 | 1 | **6** |
| **18** | Safari et al. | 2016 | 1 | 1 | 0 | 0 | 0 | 1 | 0 | 0 | 1 | 1 | **4** |
| **19** | Chen et al. | 2017 | 1 | 0 | 1 | 1 | 0 | 1 | 1 | 0 | 1 | 1 | **7** |
| **20** | Salama et al. | 2017 | 1 | 1 | 0 | 0 | 0 | 1 | 0 | 0 | 1 | 0 | **4** |
| **21** | Chung et al. | 2018 | 1 | 0 | 0 | 0 | 0 | 0 | 1 | 0 | 1 | 1 | **4** |
| **22** | Wang et al. | 2018 | 1 | 0 | 1 | 1 | 0 | 1 | 1 | 0 | 1 | 1 | **7** |
| **23** | Xue et al. | 2019 | 1 | 0 | 1 | 0 | 0 | 0 | 1 | 0 | 1 | 0 | **4** |
| **24** | Mendes-Pinheiro et al. | 2019 | 1 | 1 | 0 | 0 | 0 | 1 | 1 | 0 | 1 | 1 | **6** |
| **25** | Lee et al. | 2020 | 1 | 1 | 1 | 0 | 0 | 0 | 0 | 0 | 1 | 1 | **5** |
| **26** | Ghahari et al. | 2020 | 1 | 1 | 1 | 1 | 0 | 1 | 0 | 0 | 1 | 1 | **7** |
| **27** | Yang et al. | 2020 | 1 | 0 | 1 | 1 | 0 | 0 | 0 | 0 | 1 | 1 | **5** |

## References


Camp, D.M., Loeffler, D.A., Farrah, D.M., Borneman, J.N., and LeWitt, P.A. (2009). Cellular immune response to intrastriatally implanted allogeneic bone marrow stromal cells in a rat model of Parkinson's disease. *J Neuroinflammation* 6**,** 17. doi: 10.1186/1742-2094-6-17.

Capitelli, C.S., Lopes, C.S., Alves, A.C., Barbiero, J., Oliveira, L.F., da Silva, V.J., et al. (2014). Opposite effects of bone marrow-derived cells transplantation in MPTP-rat model of Parkinson's disease: a comparison study of mononuclear and mesenchymal stem cells. *Int J Med Sci* 11(10)**,** 1049-1064. doi: 10.7150/ijms.8182.

Cerri, S., Greco, R., Levandis, G., Ghezzi, C., Mangione, A.S., Fuzzati-Armentero, M.T., et al. (2015). Intracarotid Infusion of Mesenchymal Stem Cells in an Animal Model of Parkinson's Disease, Focusing on Cell Distribution and Neuroprotective and Behavioral Effects. *Stem Cells Transl Med* 4(9)**,** 1073-1085. doi: 10.5966/sctm.2015-0023.

Chen, D., Fu, W., Zhuang, W., Lv, C., Li, F., and Wang, X. (2017). Therapeutic effects of intranigral transplantation of mesenchymal stem cells in rat models of Parkinson's disease. *J Neurosci Res* 95(3)**,** 907-917. doi: 10.1002/jnr.23879.

Chung, T.H., Hsu, S.C., Wu, S.H., Hsiao, J.K., Lin, C.P., Yao, M., et al. (2018). Dextran-coated iron oxide nanoparticle-improved therapeutic effects of human mesenchymal stem cells in a mouse model of Parkinson's disease. *Nanoscale* 10(6)**,** 2998-3007. doi: 10.1039/c7nr06976f.

Dezawa, M., Kanno, H., Hoshino, M., Cho, H., Matsumoto, N., Itokazu, Y., et al. (2004). Specific induction of neuronal cells from bone marrow stromal cells and application for autologous transplantation. *J Clin Invest* 113(12)**,** 1701-1710. doi: 10.1172/jci20935.

Ghahari, L., Safari, M., Rahimi Jaberi, K., Jafari, B., Safari, K., and Madadian, M. (2020). Mesenchymal Stem Cells with Granulocyte Colony-Stimulating Factor Reduce Stress Oxidative Factors in Parkinson's Disease. *Iran Biomed J* 24(2)**,** 89-98. doi: 10.29252/ibj.24.2.89.

Huang, Y., Chang, C., Zhang, J., and Gao, X. (2012). Bone marrow-derived mesenchymal stem cells increase dopamine synthesis in the injured striatum. *Neural Regen Res* 7(34)**,** 2653-2662. doi: 10.3969/j.issn.1673-5374.2012.34.002.

Jadidi, M., Biat, S.M., Sameni, H.R., Safari, M., Vafaei, A.A., and Ghahari, L. (2016). Mesenchymal stem cells that located in the electromagnetic fields improves rat model of Parkinson's disease. *Iran J Basic Med Sci* 19(7)**,** 741-748.

Lee, J.Y., Kim, H.S., Kim, S.H., Kim, H.S., and Cho, B.P. (2020). Combination of Human Mesenchymal Stem Cells and Repetitive Transcranial Magnetic Stimulation Enhances Neurological Recovery of 6-Hydroxydopamine Model of Parkinsonian's Disease. *Tissue Eng Regen Med* 17(1)**,** 67-80. doi: 10.1007/s13770-019-00233-8.

Leveque, X., Mathieux, E., Nerriere-Daguin, V., Thinard, R., Kermarrec, L., Durand, T., et al. (2015). Local control of the host immune response performed with mesenchymal stem cells: perspectives for functional intracerebral xenotransplantation. *J Cell Mol Med* 19(1)**,** 124-134. doi: 10.1111/jcmm.12414.

Li, Y., Chen, J., Wang, L., Zhang, L., Lu, M., and Chopp, M. (2001). Intracerebral transplantation of bone marrow stromal cells in a 1-methyl-4-phenyl-1,2,3,6-tetrahydropyridine mouse model of Parkinson's disease. *Neurosci Lett* 316(2)**,** 67-70.

Mendes-Pinheiro, B., Anjo, S.I., Manadas, B., Da Silva, J.D., Marote, A., Behie, L.A., et al. (2019). Bone Marrow Mesenchymal Stem Cells' Secretome Exerts Neuroprotective Effects in a Parkinson's Disease Rat Model. *Front Bioeng Biotechnol* 7**,** 294. doi: 10.3389/fbioe.2019.00294.

Nezhadi, A., Ghazi, F., Rassoli, H., Bakhtiari, M., Ataiy, Z., Soleimani, S., et al. (2011). BMSC and CoQ10 improve behavioural recovery and histological outcome in rat model of Parkinson's disease. *Pathophysiology* 18(4)**,** 317-324. doi: 10.1016/j.pathophys.2011.05.004.

Offen, D., Barhum, Y., Levy, Y.S., Burshtein, A., Panet, H., Cherlow, T., et al. (2007). Intrastriatal transplantation of mouse bone marrow-derived stem cells improves motor behavior in a mouse model of Parkinson's disease. 72**,** 133-143. doi: 10.1007/978-3-211-73574-9_16.

Park, B.N., Kim, J.H., Lee, K., Park, S.H., and An, Y.S. (2015). Improved dopamine transporter binding activity after bone marrow mesenchymal stem cell transplantation in a rat model of Parkinson's disease: small animal positron emission tomography study with F-18 FP-CIT. *Eur Radiol* 25(5)**,** 1487-1496. doi: 10.1007/s00330-014-3549-3.

Safari, M., Jafari, B., Zarbakhsh, S., Sameni, H., Vafaei, A.A., Mohammadi, N.K., et al. (2016). G-CSF for mobilizing transplanted bone marrow stem cells in rat model of Parkinson's disease. *Iran J Basic Med Sci* 19(12)**,** 1318-1324. doi: 10.22038/ijbms.2016.7918.

Salama, M., Sobh, M., Emam, M., Abdalla, A., Sabry, D., El-Gamal, M., et al. (2017). Effect of intranasal stem cell administration on the nigrostriatal system in a mouse model of Parkinson's disease. *Exp Ther Med* 13(3)**,** 976-982. doi: 10.3892/etm.2017.4073.

Shetty, P., Ravindran, G., Sarang, S., Thakur, A.M., Rao, H.S., and Viswanathan, C. (2009). Clinical grade mesenchymal stem cells transdifferentiated under xenofree conditions alleviates motor deficiencies in a rat model of Parkinson's disease. *Cell Biol Int* 33(8)**,** 830-838. doi: 10.1016/j.cellbi.2009.05.002.

Suzuki, S., Kawamata, J., Iwahara, N., Matsumura, A., Hisahara, S., Matsushita, T., et al. (2015). Intravenous mesenchymal stem cell administration exhibits therapeutic effects against 6-hydroxydopamine-induced dopaminergic neurodegeneration and glial activation in rats. *Neurosci Lett* 584**,** 276-281. doi: 10.1016/j.neulet.2014.10.039.

Wang, T.H., Feng, Z.T., Wei, P., Li, H., Shi, Z.J., and Li, L.Y. (2008). Effects of pcDNA3-beta-NGF gene-modified BMSC on the rat model of Parkinson's disease. *J Mol Neurosci* 35(2)**,** 161-169. doi: 10.1007/s12031-007-9032-8.

Wang, X., Zhuang, W., Fu, W., Wang, X., Lv, E., Li, F., et al. (2018). The lentiviral-mediated Nurr1 genetic engineering mesenchymal stem cells protect dopaminergic neurons in a rat model of Parkinson's disease. *Am J Transl Res* 10(6)**,** 1583-1599.

Xiong, N., Yang, H., Liu, L., Xiong, J., Zhang, Z., Zhang, X., et al. (2013). bFGF promotes the differentiation and effectiveness of human bone marrow mesenchymal stem cells in a rotenone model for Parkinson's disease. *Environ Toxicol Pharmacol* 36(2)**,** 411-422. doi: 10.1016/j.etap.2013.05.005.

Xue, J., Liu, Y., Darabi, M.A., Tu, G., Huang, L., Ying, L., et al. (2019). An injectable conductive Gelatin-PANI hydrogel system serves as a promising carrier to deliver BMSCs for Parkinson's disease treatment. *Mater Sci Eng C Mater Biol Appl* 100**,** 584-597. doi: 10.1016/j.msec.2019.03.024.

Yang, C., Qiu, Y., Qing, Y., Xu, J., Dai, W., Hu, X., et al. (2020). Synergistic effect of electric stimulation and mesenchymal stem cells against Parkinson's disease. *Aging (Albany NY)* 12(16)**,** 16062-16071. doi: 10.18632/aging.103477.

Ye, M., Wang, X.J., Zhang, Y.H., Lu, G.Q., Liang, L., Xu, J.Y., et al. (2007). Therapeutic effects of differentiated bone marrow stromal cell transplantation on rat models of Parkinson's disease. *Parkinsonism Relat Disord* 13(1)**,** 44-49. doi: 10.1016/j.parkreldis.2006.07.013.

Yin, X., Xu, H., Jiang, Y., Deng, W., Wu, Z., Xiang, H., et al. (2014). The effect of lentivirus-mediated PSPN genetic engineering bone marrow mesenchymal stem cells on Parkinson's disease rat model. *PLoS One* 9(8)**,** e105118. doi: 10.1371/journal.pone.0105118.
